# ﻿Revision of the family Milacidae from Switzerland (Mollusca, Eupulmonata, Parmacelloidea)

**DOI:** 10.3897/zookeys.1116.82762

**Published:** 2022-08-09

**Authors:** Vivianne M. Schallenberg, René Heim, Ulrich E. Schneppat, Peter Müller, Jörg Rüetschi, Eike Neubert

**Affiliations:** 1 Natural History Museum Bern, Bernastrasse 15, CH-3005 Bern, Switzerland; 2 Institute of Ecology and Evolution, University of Bern, CH-3012 Bern, Switzerland; 3 St.-Karli-Strasse 32c, CH-6004 Lucerne, Switzerland; 4 Natur Museum Lucerne, Kasernenplatz 6, CH-6003 Lucerne, Switzerland; 5 Sennereiweg 8, CH-7074 Churwalden-Malix, Switzerland; 6 Witikonerstrasse 180, CH-8053 Zurich, Switzerland; 7 Malakologische Untersuchungen, Weidweg 42, CH-3032 Hinterkappelen, Switzerland

**Keywords:** barcoding, colour variation, distribution records, genital organs, *Milax*, slugs, *Tandonia*

## Abstract

In this work, the presence of species of the slug family Milacidae in Switzerland was investigated by using the barcoding marker cytochrome c oxidase subunit I (**COI**) as well as traits of the body and the genital organs. Currently, three species of *Tandonia* living in Switzerland in established populations could be reported, i.e., *T.rustica*, *T.budapestensis*, and *T.nigra*. The three records of *Milaxgagates* were re-investigated, but only for one of these records could the identification be reconfirmed. This species has currently no established and thriving population in Switzerland. For all species recorded, detailed descriptions of body morphology, genital anatomy, and distribution data are provided based on the investigated Swiss animals. An unknown pale colour morph of a *Tandonia* sp. from Canton Ticino could be identified as *T.nigra*, and the barcodes of *T.nigra* specimens were submitted to GenBank for the first time. The identity of the Italian and Austrian populations of *T.nigra* from the Bergamasque Alps and north Tirol is evaluated. Observations on details of the morphology of the genital organs in *T.rustica* vs. *T.kusceri* are discussed.

## ﻿Introduction

The family Milacidae Ellis, 1926 currently comprises two genera with 14 extant species in *Milax* Gray, 1855, and 29 extant species in *Tandonia* Lessona & Pollonera, 1882 (MolluscaBase, accessed on 28 December 2021). The last comprehensive revision of the family was published by Wiktor (1987). The family is distributed in Europe with the Balkan peninsula as the area of highest species richness. In Switzerland, the family is represented by only four species (Bank and Neubert 2017). These species were listed in the Red List for continental molluscs of Switzerland, and their conservation status was assessed (Rüetschi et al. 2012). According to these lists (Rüetschi et al. 2012; Bank and Neubert 2017), the following milacid species are known from Switzerland: *Milaxgagates* (Draparnaud, 1801), *Tandoniabudapestensis* (Hazay, 1880), *Tandoniarustica* (Millet, 1843), and *Tandonianigra* (C. Pfeiffer, 1894).

*Tandoniarustica* is a rarely discovered but widespread taxon in the country; it is a nocturnal species strictly confined to beech forests on limestone talus (pers. obs.). The species is also recorded from the Italian Alps ranging from Canton Ticino to Lake Garda and from many locations in the Piemontese Alps in Valle di Susa (Wiktor 1987; Gavetti et al. 2008; pers. obs.). In contrast, *T.nigra* is considered to represent a rather local alpine species with a smaller distribution area. The type locality of this species is the summit of Monte Generoso in Canton Ticino close to Lugano. According to Wiktor (1987), the species is also recorded from the Austrian Alps in North Tirol, and the recent Red List assessment by the International Union for Conservation of Nature (**IUCN**) records a distribution from the Italian Alps ranging from the Canton Ticino to Lake Garda (Nardi 2017). The identity of these Austrian and Italian records is discussed below. In the last years, one of the authors (P. Müller) observed a form of a small *Tandonia* species in Val Cürta on the southwestern slope of the Monte Generoso, which differed from the black *T.nigra* from the summit by a grey to beige body colour and an elongate very slim penial papilla.

The aim of this paper is to verify the correct identification of the *Tandonia* species from Switzerland in general, and to specifically investigate the status of the grey to beige *Tandonia* specimens from lower altitudes of Monte Generoso. We compared our genetic data with a few species recorded in GenBank by Liberto et al. 2012; Rowson et al. 2014a; Korábek et al. 2016; Turóci et al. 2020 and other unpublished data to corroborate our identifications. Since the results of the COI analysis produced well-supported data, we decided not to analyse further markers. Our aim was to characterise species with a simple marker, and not to reveal phylogenetic relationships of the clades detected, which would have required a much deeper research approach with a sampling that exceeds our current possibilities.

## ﻿Materials and methods

### ﻿Specimens

Most investigated specimens were freshly collected during field trips in Switzerland, Italy, Republic of San Marino, and France. If possible and necessary, juveniles were kept alive until they reached adulthood. To complete and increase the genetic data set also previously inventoried specimens of the Natural History Museum Bern (**NMBE**) were included in this study. Detailed sampling locations, voucher numbers and the GenBank accession numbers of the sequenced specimens are shown in Suppl. material 1: Table S1. All records in this study will be uploaded to the map server of the Centre Suisse de Cartographie de la Faune (**CSCF**) and will be displayed on the respective species distribution maps (https://lepus.unine.ch/carto/).

### ﻿Husbandry of live slugs and their preservation

Juvenile and subadult slugs were kept individually in 0.2 L polypropylene boxes (Rondo 4, Rotho Kunststoff AG, Switzerland) perforated with a hot needle for air exchange. The boxes were stored in a wine refrigerator at 16 °C, the bottom was carpeted with several layers of moistened paper towels. Nutrition was provided in form of slices of champignon, cucumber, and high protein cat feed pellets. The slugs were checked and weighed once per week with replacement of the paper towel and food. The maturity was checked by evaluation of the genital pore: fully adult animals have a visible and clearly open pore. All captured adult specimens were photographed alive, and tissue samples and body measurements were taken before preservation. For preservation, the animals were relaxed and killed in sparkling water for ~ 30 min. The dead animals were placed on kitchen paper and frozen in the freezer for several hours. After that they were thawed in cold water and additional slime was removed by placing the specimens into ethanol. The animals were transferred into 96% ethanol, and, for proper body cavity preservation, 96% ethanol was injected through the sole tip into the body cavity with a 1 mL syringe using a fine needle (Terumo Corporation, Philippines). After 24 h, the slugs were transferred in 80% ethanol. The following day, the ethanol was exchanged, and the specimens were stored in the fridge at 5 °C awaiting DNA extraction and dissection. This procedure properly maintains the soft tissue for anatomical studies and keeps the DNA intact for the genetic investigation.

### ﻿Morphological and anatomical analysis

All specimens used for live body measurements were fully adult specimen from the collection of R. Heim. The following measurements were taken:

Live weight (**lw**) was taken with a digital scale to 0.01 g weekly. The last measured weight before preservation of each specimen was taken for calculation. In the analysis the weights were rounded up to 0.5 g.

Total length (**tl**) was measured with a metal ruler from specimens in full stretch when crawling to 1 mm. Starting from the front to the outermost extension of the posterior sole or dorsum.

Sole width (**sw**) was measured from the crawling specimen on a sheet of glass with a ruler or a calliper to 0.5 mm. The widest extension was usually at mid of the sole.

The ratio of mantle length (**ml**) / total length (**tl**) is given because it might be an additional character for species differentiation.

Number of tubercle rows (**tr**) were counted on the freshly preserved animals with a dissection needle under a binocular. Starting from the first tr at the slit of the pneumostome along the posterior edge of the mantle to the last tr at the keel. Counting was done twice, back and forth. If not clearly countable, the two closest numbers were indicated. In the analysis the lowest and highest counts of our specimens were taken.

**Figure 1.**
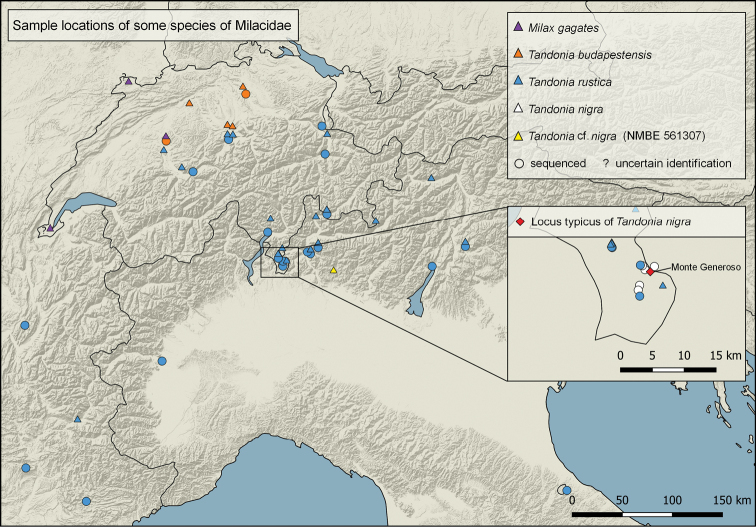
Distribution map of collected specimens.

Before dissection of selected specimens, photographs of the preserved animals were made in dorsal, lateral (left and right), and ventral position. The dissection of the slug genitalia was performed under a binocular (Leica MZ6) using thin forceps (Dumont 0.5; 3.5) and micro scissors. For the anatomical pictures, the genital organs were detached from the body, spread on a wax bed, and properly pinned with minutia pins to visualize and investigate the structures. Additionally, the inner structures of penis, epiphallus and vagina were shown. Pictures were taken with a Leica DVM6 microscope camera (Leica Camera, Wetzlar, Germany) using the image-processing program FIJI for scaling (Schindelin et al. 2012). The distribution map of the investigated species (Fig. 1) was created using QGIS software v3.20.3-Odense (QGIS.org, 2022. QGIS Geographic Information System. QGIS Association. http://www.qgis.org).

### ﻿Molecular analysis

The total DNA extraction was performed following the manufacturers protocol of the Qiagen Blood and Tissue Kit using the QIAcube extraction robot (Qiagen; Hilden, Germany). Approximately 0.5 cm^2^ of mantle tissue was cut, cleaned with a sterilized scalpel (Schreiber GmbH, Germany) from superficial slime, and placed in 180 µL ATL buffer and 20 µL proteinase K. For small specimen, an additional snippet from the foot or body wall was added to the digestion mix in order to yield enough DNA for sequencing. The tissue was then incubated for 4 h at 56 °C and 40 rpm in a thermo shaker (Labnet, Vortemp 56, witec AG, Littau, Switzerland). After digestion the QIAcube extraction robot did the DNA extraction following the standard protocol 430 (DNeasy Blood Tissue and Rodent tails Standard). The extracted DNA samples were then stored in a -80 °C freezer for long-term storage.

In this study, the sequences of the mitochondrially encoded barcoding marker cytochrome c oxidase I (**COI**) was investigated. For the COIPCR mixture 3 µL of DNA template in 12.5 µL GoTaq G2 HotStart Green Master Mix (Promega M7423), 7.5 µL ddH2O, 1 µL of the established Folmer primers LCO1490 and HCO2198 (Folmer et al. 1994) were used. The PCR cycle was set as following: 3 min at 94 °C, followed by 40 cycles of 1 min at 95 °C, 1 min at 47 °C and 1 min at 72 °C and for the last step, the final elongation 10 min at 72 °C. The PCR products were controlled using gel electrophoresis. PCR products with a visible, single band with the desired length were sent to LGC (LGC Genomics Berlin, Germany) for purification and Sanger sequencing. For each specimen, the forward and reverse sequence were aligned using the software package Geneious v. 9.1.8 (Biomatters Ltd) and the consensus sequence was extracted. Finally, the consensus sequence was trimmed to the 655 bp used in further analysis.

### ﻿Phylogenetic analysis

For the phylogenetic analyses, sequence KT371419 from GenBank (*Oxychilusdraparnaudi* (H. Beck, 1837)) was included as outgroup. Additionally, 34 sequences were downloaded from GenBank comprising *Tandoniabudapestensis*, *Tandoniarustica*, *Tandoniakusceri* (H. Wagner, 1931), *Tandoniasowerbyi* (A. Férussac, 1823) and *Tandoniacristata* (Kaleniczenko, 1851). The sequences were included into the phylogenetic analysis to obtain a better overview of the genus *Tandonia* and to compare our results to those of Rowson et al. (2014a). The included species and their accession numbers are listed in the Suppl. material 1: Table S1.

For sequence processing, alignments and calculation of trees, the software package Geneious v. 9.1.8 (Biomatters Ltd) was used. The protein-coding gene fragment of COI was defined in three data blocks, with each codon position as separate subset.

To calculate the ML interference, the RAxML plug-in for Geneious (Stamatakis 2006) was implemented, using the Geneious plug-in with rapid bootstrapping setting, the search for the best scoring ML tree, the nucleotide model GTR CAT I and 2000 bootstrapping replicates.

With the same alignment data, a second maximum likelihood tree was calculated using the IQ Tree web tool (http://iqtree.cibiv.univie.ac.at/) (Trifinopoulos et al. 2016), using the default settings for the input data and the substitution model set to auto detect. The branch support analysis was set to ultrafast with 1000 bootstrap alignment and performing a SH-aLRT branch test (Guindon et al. 2010) with a 1000 replicates.

Bayesian Inference (**BI**) was performed using Mr. Bayes v3.2.6 × 64 (Huelsenbeck and Ronquist 2001; Ronquist and Huelsenbeck 2003; Altekar et al. 2004) through the HPC cluster from the University of Bern (http://www.id.unibe.ch/hpc). For the data set, the nucleotide model was set 4 by 4, and we applied a mixed model search. The Monte Carlo Markov Chain (MCMC) parameter was set as follows: starting with four chains and four separate runs for 15 million generations with the temperature set to 0.2. The tree sampling frequency was set to 1000 with a burn in of 25%.

### ﻿Species delimitation analysis

Species partitions analysis was performed on the Assemble Species by Automatic Partitioning (**ASAP**) web tool (https://bioinfo.mnhn.fr/abi/public/asap/asapweb.html) (Puillandre et al. 2021), using the Kimura (K80) (Kimura 1980) substitution model with the default settings for the transition/transversion rate ratio (ts/tv) = 2 and the standard advanced options for 5 best partition suggestions for 71 available COI sequences.

### ﻿Abbreviations

**ad** adult

**ASAP** Assemble Species by Automatic Partitioning

**BI** Bayesian Interference

**BS** Bootstrap support

**COI** cytorchrome c oxidase subunit I

**CSCF** Centre Suisse de Cartographie de la Faune (Neuchâtel, Switzerland)

**gp** genital pore

**HNHM** Hungarian Natural History Museum, Budapest

**IUCN** International Union for Conservation of Nature

**juv** juvenile

**lw** live weight

**MHNG** Muséum d’histoire naturelle de la Ville de Genève

**ml** mantle length

**ML** maximum likelihood

**nc** not countable

**NMB** Natural History Museum Basel

**NMBE** Natural History Museum Bern

**NMLU** Natur-Museum Luzern

**NMW** National Museum of Wales, Cardiff

**PCR** Polymerase Chain Reaction

**SMNS** State Museum of Natural History Stuttgart

**sw** sole width

**tl** total length

**tr** tubercle rows

**ZMH** Zoological Museum Hamburg

## ﻿Results

**Figure 2.**
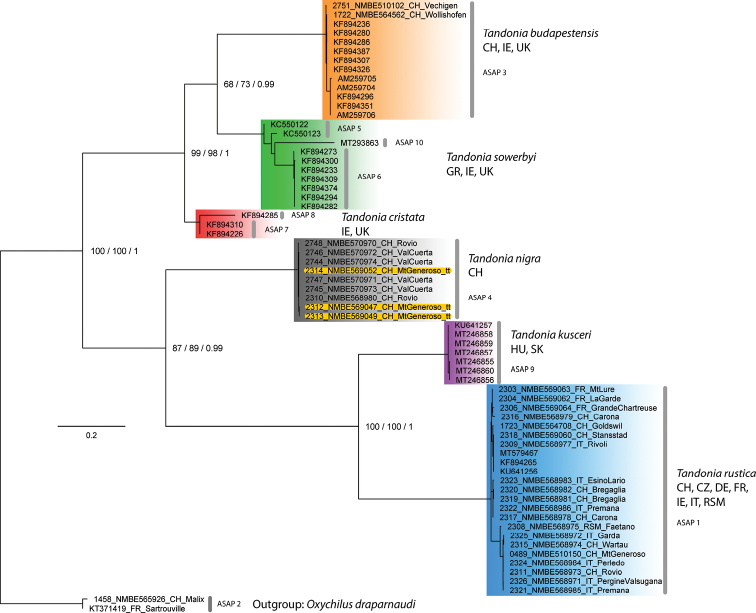
Phylogenetic tree of some species of the genus *Tandonia* based on COI. Numbers at the nodes record support values for RaxML/IQTree/BI. Sequences published in GenBank are named with their accession number; new sequences obtained are named as following: Lab number, museums voucher number, country, and approximate locality. The grey vertical bars indicate the best score for species delimitation calculated with ASAP.

The results of the genetic analysis based on COI are summarised in Fig. 2. The tree is based on the topology of RaxML, which was confirmed by IQTree and Bayesian Interference. Interpretation of Bootstrap values for RaxML and IQ Tree: 70 to 80 = moderate support; 80 to 90 = well supported; > 90 = high support and for the Bayesian posterior probabilities values: > 0.95 = significant support. This tree does not reflect relationship of species as only six out of 29 currently accepted species are included (https://molluscabase.org/aphia.php?p=taxdetails&id=819994 on 28 December 2021); the selection of taxa mainly followed the availability of COI sequences.

*Tandonianigra* appears as a rather well supported clade within the selected group. The three highlighted sequences originate from topotypic specimens from the top of Monte Generoso. All other populations investigated were collected from a narrow range at lower altitudes on the mountain and contain all the different colour morphs. Despite their differing body colour (Fig. 8), there is almost no variation in the COI sequences of these specimens observable. Furthermore, the genetic clades of *T.rustica* and *T.kusceri* are clearly separated and well supported.

The best two ASAP scores support the existence of ten different clades as visualized in Fig. 2 with the grey bars labelled with numbers from 1 to 10. This gives 3 subclades in the *T.sowerbyi* clade and two for the *T.cristata* clade. The third best score however supports the concept of seven different clades in the same way, as the clades in Fig. 2 are coloured.

### ﻿Taxonomy

#### ﻿ Milacidae

Taxon classificationAnimaliaStylommatophoraMilacidae

Ellis, 1926

96DDB378-7F64-5A27-8591-C46A373A4F9E

##### Diagnostic features.

Horse-shoe shaped groove on the mantle; pneumostome postmedian; keel connecting sole tip and mantle; sole tripartite, central sole field with V-shaped wrinkles, only visible in preserved animals (Wiktor 1987).

#### ﻿ Tandonia

Taxon classificationAnimaliaStylommatophoraMilacidae

Lessona & Pollonera, 1882

219F3258-F686-50D3-8DDC-4A914118E94D

##### Diagnostic features.

No stimulatory organ in the atrium, accessory glands opening into the vagina (Wiktor 1987).

#### ﻿ Tandonia rustica

Taxon classificationAnimaliaStylommatophoraMilacidae

(Millet, 1843)

A19686D8-31A3-53DB-BFC9-397668CD49AC

Figs 1 , 3 , 7A–D

Limax rustica Millet, 1843, Magasin de Zoologie, ser. 2, 5: 1, plate 63, fig. 1 [nord de l’Anjou, à la Bouillant, commune de la Chapelle-Hullin, ainsi qu’à Thorigné, etc.].

##### Type specimens.

Probably does not exist, after Wiktor (1987: 286). See also remarks.

##### Differential diagnosis.

Epiphallus more than twice as long as the penis (almost the same length in *T.nigra* and *T.budapestensis*); dark stripe above the pneumostome and on the opposite side of the mantle (missing in *T.nigra* and *T.budapestensis*); penial papilla short, blunt, with flap-like lobes around the central porus (short and blunt but folded in *T.nigra*; elongated with a stalk in *T.budapestensis*).

##### Description.

***Colouration*.** Many fully adult specimens (*n* = 56) were studied from six different Swiss Cantons (Bern, St Gallen, Grisons, Ticino, Obwalden, Nidwalden) as well as from five regions in Italy (Bolzano, Sondrio, Torino, Lecco, Trento), from the Republic of San Marino and from two Departments of France (Alpes de Haute Provence, Isère).

The general pigmentation of Swiss specimens varies from a warm reddish brown to dark reddish or chestnut brown to very dark brown without any reddish brown hue. This pigmentation normally is not fading downwards to the fringe of the sole. However, in some cases in the less dark pigmented populations in Switzerland, little fading downwards can be observed (Fig. 7A). The mantle is of the same colour as the dorsum, except for the surrounding of the pneumostome, which usually is paler. Many specimens investigated are markedly darker when compared to Wiktor (1973, 1987) and Rowson et al. (2014b). The darkest specimens were found in southern Switzerland (Ticino, Grisons - Val Bregaglia), and in the southwest bordering areas to Italy and France (Fig. 7B). Specimens from Central Switzerland (Lucerne, Nidwalden, Obwalden), northern and eastern Grisons, St. Gallen, and the eastern border area in Italy (Bolzano, Trento) are of a markedly lighter, pale reddish brown pigmentation (Fig. 7A, C, D).

The whole slug is covered with small black spots on dorsum, flanks, and mantle, but not on the keel. The characteristic black streaks above the pneumostome and on the same location on the left side of the mantle can vary greatly in size and form. Even though all our investigated specimens had spots and the characteristic streaks on the mantle, in some specimens they are almost invisible because of the overall dark pigmentation (Fig. 7B). Additionally spots in darker specimen may be much larger and sometimes irregularly jagged and elongated to streaks.

Head, neck and ommatophores are dark brown, while the tentacles are slightly paler. In many cases, the black pigmented ommatophoran retractor is clearly visible through the integument.

The keel is commonly paler in colour than the dorsum. Rarely the general pigmentation of the keel matches the dorsal colour, this occurs especially in darker specimens.

The tripartite sole in pale coloured slugs is creamy yellowish, while darker slugs have a pale yellowish brown sole. Many darker coloured specimens have a hue of greyish black at the posterior outer margins of the lateral sole fields formed by very little black dots. Isolated single greyish black spots may occur along the outer edge of the sole.

***Mantle structure* .** The pneumostome is positioned at 2/3 of mantle length, well posterior of the centre of the mantle. The pneumostome is not surrounded by a distinct ring-like structure, like it is the case in *T.nigra*. The “slit” of the pneumostome in all specimens does not end in the lumen of the pneumostome but runs anteriorly to at least the dorsal edge of pneumostome. In living individuals, the mantle surface is completely smooth besides the horseshoe-shaped sinus groove. The sinus groove is completely developed in all specimens in our series, and it reaches at both sides almost the posterior end of the mantle. In the dark specimens it needs magnification to see it clearly. The posterior margin of the mantle is not tightly attached to the integument and in living and contracted animals smoothly rounded. The posterior free mantle flap covers the anterior integument-tubercles as well as the openings of the postpallial or Wiktor’s pocket organ.

***Postpallial pocket organ*.** In all specimens examined, the posterior part of the mantle covered two slit-like openings, the postpallial pocket organ, which was first detected and described by Schneppat et al. (2011) for *Tandoniatotevi* (Wiktor 1975).

***Integument structures*.** The number of tr (*n* = 52; tr 12/13-tr 19, ø tr 15) does not vary much in our specimens, and there is no significant variation between populations. The surface texture and the width of tubercles in live specimens vary from fully straight to torn, depending on their body position. All tr from head to the 7^th^–9^th^ row posterior to the pneumostome are entirely flat, smooth, wide, and remain so from the head and flank to the peripodial tubercle. The remaining tr reaching to the keel are somewhat crenulated, but are also wide, flat, and lacking ridges. Crenulations can only be seen under magnification. The tubercle rows may be long and undivided down to the peripodial tubercle, especially the ones below the lateral mantle edge. The more dorsal rows usually are divided in several tubercle compartments.

The keel is extended from the posterior margin of the mantle to the end of the dorsum. In some specimens, the keel was observed to be even slightly extending over the fringe of the sole, like a very little terminal thorn or knob. The keel has always an entirely smooth surface structure and is therefore clearly discernible from the crenulated neighbouring dorsal tubercles. Live and preserved specimens do not differ in the extension of the keel and its structure.

***Sole structure*.** The outermost edge or seam of the sole is separated from the dorsum by a longitudinal fold, the peripodial tubercle, which begins left and right of the mouth-flaps and runs posterior around the body. The peripodial tubercle together with the peripodial groove clearly separate the seam of the sole at its outermost posterior end, where the sole is rounded and not pointed. In the central field of the sole, many V-shaped transverse wrinkles exist, which are invisible in animal crawling on a pane of glass but are well visible in preserved specimens.

***Mucus*.** The mucus lacks any pigmentation on body, mantle and sole and is extremely sticky. When irritated, some of the animals produced defensive mucus of white to yellowish greenish colour on dorsum, flanks, and mantle, but this could only rarely be observed.

***Measurements*.**lw (*n* = 56): 1.5–6 g, ø 3 g; tl (*n* = 56): 52–92 mm, ø 69 mm; ml (*n* = 50): 18–33 mm, ø 23 mm; sw (*n* = 47): 5–10 mm, ø 7.3 mm. Ratio of tl/ml ranges from ca. 52/18 mm to ca. 92/33 mm; ø ml is 1/3 of tl.

**Figure 3.**
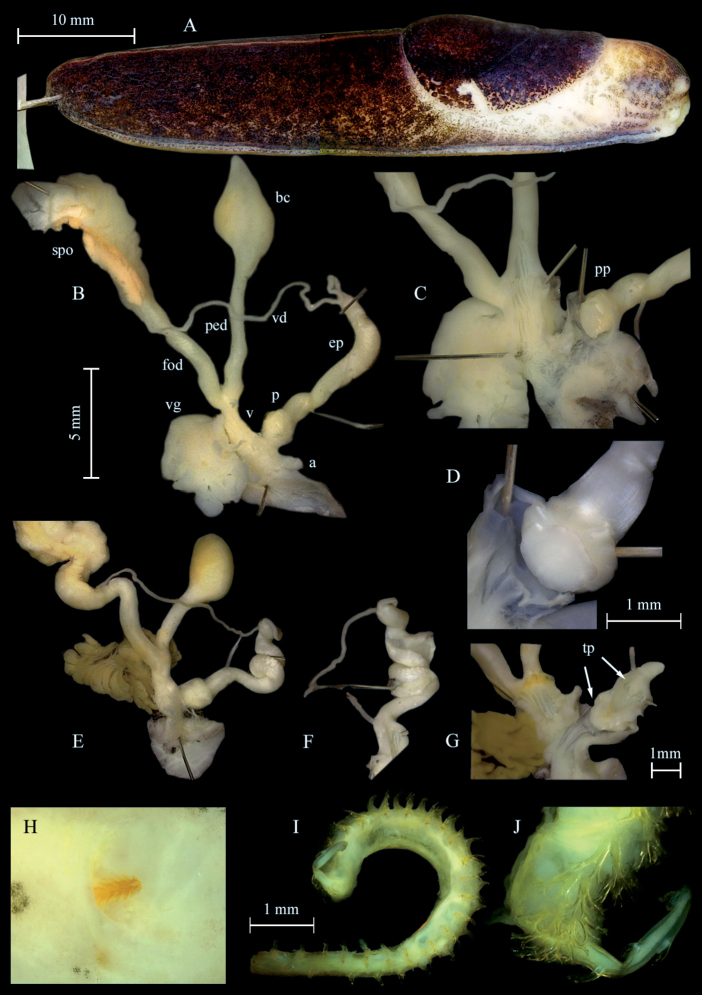
*Tandoniarustica.***A–C** sequenced specimen NMBE 568973, Rovio, Sovaglia **A** preserved animal **B** situs of genitalia **C** distal genitalia showing anatomical details **D** penis papillae of sequenced specimen NMBE 568978, Carona **E–G** sequenced specimen NMBE 564708, Goldswil **E** situs of genitalia **F** distorted epiphallus **G** details of lumina of vagina, atrium and penis, arrows pointing at the “twin papillae” (tp) **H–J** spermathophore of specimen NMBE 571414, Orselina **H** genital pore with piece of spermatophore **I, J** largest part of the three pieces **J** detail of surface covering spines. Abbreviations: a = atrium; bc = bursa copulatrix; ep = epiphallus; fod = free oviduct; p = penis; ped = pedunculus; pp = penial papilla; spo = spermoviduct; tp = “twin papillae”; v = vagina; vd = vas deferens; vg = vaginal gland.

***Genital organs*.** Atrium very short and tubular; penis short, with a distal bulb harbouring the penial papilla and a second bulb consisting of the papilla basis marking the boundary to the epiphallus, interior penial walls simple; penis papilla ornamented, apex of papilla with a row of curved crests encircling the complete papilla giving it a flower like appearance (Fig. 3C, F); penis retractor muscle inserting at the epiphallus/penis boundary; epiphallus externally with smooth surface, consistent in diameter, reaching up to 4 times the length of the penis.

Vagina twice the length of the penis, separated from the atrium by a sphincter; accessory glands entering close to the boundary of atrium and vagina; accessory glands digitiform or sac-like, either beige, brown or bright rusty red coloured; vaginal lumen with elongated, waved folds pointing towards the oviduct and the pedunculus of the bursa copulatrix; pedunculus somewhat longer than the vesicle; vesicle may be pointed or rounded.

***Spermatophore***. A spermatophore was found in a single specimen, broken into three parts (NMBE 571414, Ticino, Orselina, 14 February 2020). One part still stuck in the lumen of the genital pore of the slug, length 0.2 cm; it is completely covered with simple spines (Fig. 3H). The other two parts were found embedded in the vesicle in a white, fibrous mass; digestion had already started. The smaller part is 0.35 cm long, curved and covered with undivided and, further on, bifurcated spines laterally. The largest part of the spermatophore is ~ 0.9 cm long and curled (visualized in Fig. 3I, J). Its outer surface is covered on the entire length with bifurcated spines. This is the first record of the spermatophore of this species.

##### Distribution.

Our own distribution records are mainly limited to Switzerland, France, northern Italy, and the Republic of San Marino. The species itself is frequently recorded from the western alpine arc, but also from a wide range in Great Britain, Ireland, The Netherlands, Belgium, Luxemburg, France, Germany, Austria, Poland, Czech Republic, Slovakia, and Hungary (Wiktor 1987; Gavetti et al. 2008; Rowson 2017). Reports for Romania are likely misidentifications with *T.kusceri*. Old reports for Bulgaria are shown to be wrong identifications of the very common *T.kusceri* (pers. obs.).

##### Habitat.

This species is confined to beech forests on limestone talus.

##### Remarks.

As original name of this species, Wiktor (1987) referenced a “*Limaxmarginatusrusticus* Millet, 1843”, which is wrong. In his text, Millet states “Je l’ai rencontrée au nord de l’Anjou, à la Bouillant, commune de la Chapelle-Hullin, ainsi qu’à Thorigné, etc.”. These localities can be found in the larger surrounding north of Angers, Region Pays de la Loire, Déptartement Maine-et-Loire, thus we consider this area the “type area” of *L.rusticus*. A neotype should be selected from one of the mentioned places to unambiguously fix the use of this name.

When it comes to morphology Wiktor (1987) mentioned 18–19 tr, which is higher than most of our counts, but still in the range of our data. Comparing the exterior of living and preserved specimens with the description by Wiktor (1987: 286) and Rowson et al (2014b), many differences were found. No specimens of generally “white or white creamy” pigmentation, nor those of “pinkish or violetish” hues could be recorded by us in Switzerland and bordering areas. Most specimens investigated are explicitly darker than described by Wiktor (1987) and Rowson et al (2014b) indicating a much higher intraspecific variability of the species in other areas of distribution. Colour morphs exclude each other’s following the geographical pattern as described above. In Wiktor (1987: figs 196, 197), the pneumostome is positioned on the left side of the body, which most probably is an error or a sinistral specimen.

Regarding the genital anatomy we found genetically identified *T.rustica* (NMBE 564708, NMBE 568978), which show a very similar anatomy to *T.kusceri* with a long (in one specimen) coiled epiphallus and in both a round bursa copulatrix (Fig. 3E, F). For the coiled epiphallus it might be a sexually active specimen, very probably after the ejection of the spermatophore. Even though the characters of *T.rustica* and *T.kusceri* seem to meet, the two species can be distinguished by their habitat. *Tandoniakusceri* usually is found in disturbed, synanthropic habitats, whereas *T.rustica* is usually found in untouched more natural woodland habitats.

Wiktor (1973) and Likharev and Wiktor (1980) report a long stem of the penial papilla, an observation we could not reproduce. Later, Wiktor (1987) reported a slightly reduced length, but even this reduction does not fit to our results. In all our dissected specimen, we had a bulky papilla tightly attached to the constriction of the epiphallus and therefore without a stem.

Gerhardt (1940) observed the copulation behaviour of the species in his laboratory; however, he did not illustrate the spermatophore. He observed the first copulation on 17 November 1938 followed by two copulations on the 4 December 1938. His specimens were collected near Porto d’Ischia, Isola d’Ischia, Italy. It remains unclear whether these specimens were *T.rustica*; later research on the island yielded no specimens of this species. In case Gerhard’s determination was correct, the data of the observed copulation and those from our collection with the spermatophore found on 14 February 2020 indicate that this species probably mates during the winter season, a period where malacological research activity is rather low.

#### ﻿ Tandonia nigra

Taxon classificationAnimaliaStylommatophoraMilacidae

(C. Pfeiffer, 1894)

FCC57D1D-588B-5F64-B4DA-EEA2F14E7898

**Figure 4.**
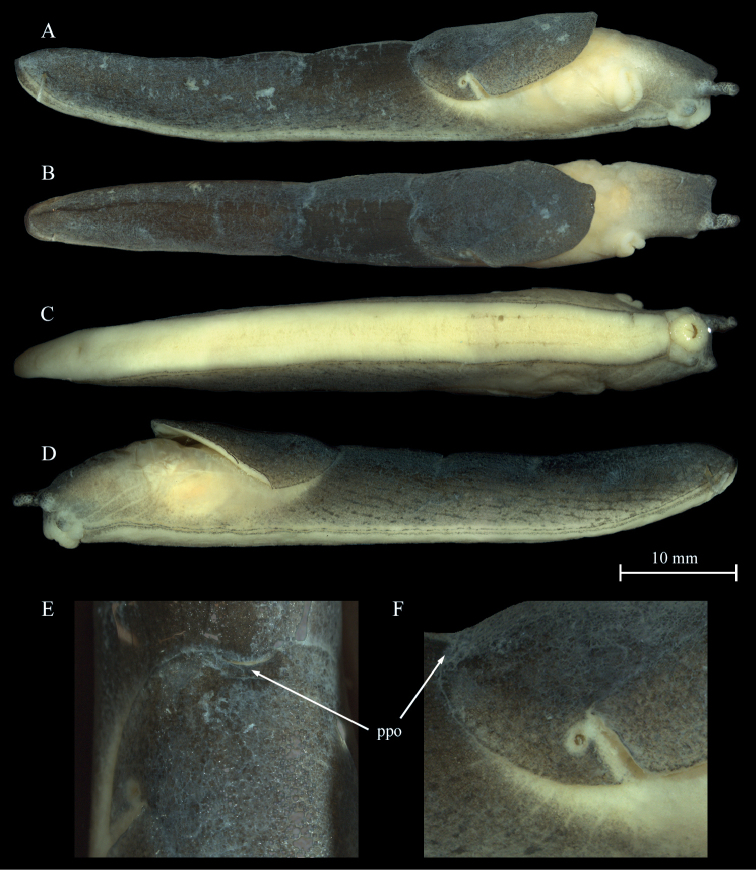
*Tandonianigra*. **A–F** sequenced topotype NMBE 569047, Monte Generoso, Rovio, Calvagione, 1.650 m alt **A** lateral view right **B** dorsal view **C** ventral view **D** lateral view left **E** dorsal view showing the postpallial pocket organ (indicated with arrows) **F** lateral right view of mantel with pneumostome. Abbreviation: ppo = postpallial pocket organ.

**Figure 5.**
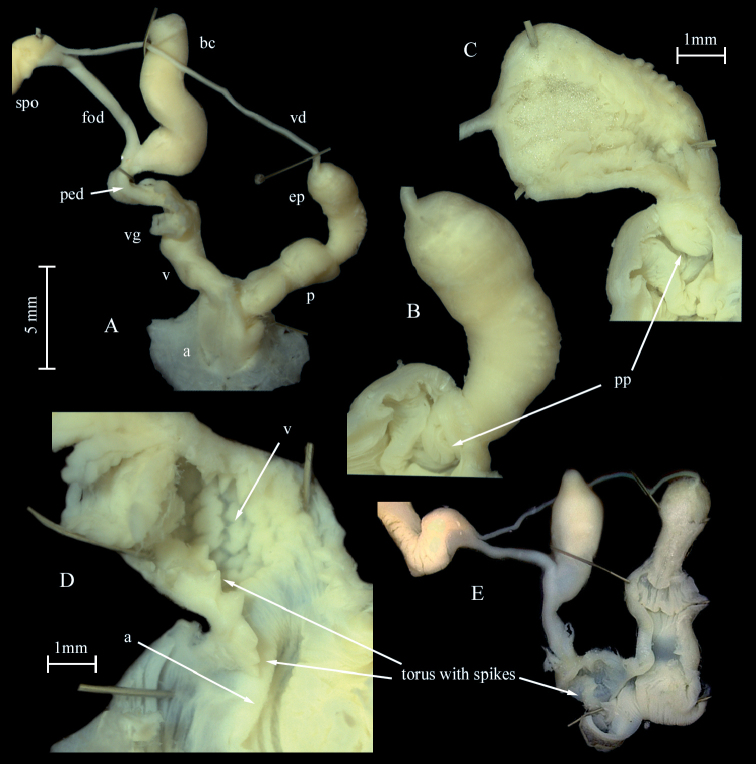
*Tandonianigra*. **A–D** sequenced topotype NMBE 569047, genital organs **A** situs of genitalia **B** penis with epiphallus showing the granulation on the epiphallial surface and the penis papilla **C** epiphallus opened to show the transparent globuli **D** vagina opened showing the torus with the row of spikes **E** sequenced specimen NMBE 568980, situs of genitalia. Abbreviations: a = atrium; bc = bursa copulatrix; ep = epiphallus; fod = free oviduct; p = penis; ped = pedunculus; pp = penial papilla; spo = spermoviduct; v = vagina; vd = vas deferens; vg = vaginal gland.

Figs 4 , 5 , 7C, D , 8

Amalia nigra C. Pfeiffer, 1894, Nachrichtsblatt der Deutschen Malakozoologischen Gesellschaft, 26: 68 [Gipfel des Mte. Generoso (1695 m.)].

##### Type specimen.

***Holotype*** SMF 107558.

##### Differential diagnosis.

Torus with spikes inside the vagina, epiphallus with a field of nodes on the surface; for other character states, refer to the paragraph under *T.rustica*.

##### Description.

***Colouration*.** The animals at the type locality are dark blackish brown coloured with the dorsum almost black (Figs 4, 8B, C). In lower altitudes of the mountain, animals were dark grey (Fig. 8A) to almost white in colour, with the mantle and dorsum finely blotched (Fig. 8D, E). Two animals also had a light greyish cream ground colouration, one with darker small grey blotches all over the body (Fig. 8F).

The mantle generally matches the dorsal colour. For the topotypic specimen, dots at the edges of the mantle are lacking. However, in light colour morphs, the mantle appears slightly darker than the dorsum because of the accumulated dots and blotches. In many of these specimens the highest density is along the sinus groove.

For the topotypical colour morph the flanks are pigmented as the dorsum, but with a narrow, paler greyish stripe just above the edge of the sole with little blackish grey dots and stripes. This also refers to the flanks below the edge of mantle. In the light colour morphs, the dorsum and flanks are covered by very fine dark dots on the top of the tubercles (not along the grove lines; not a reticulation). This pigmentation pattern is found from the dorsum down to the edge of the sole. Some larger, irregularly scattered black spots more posterior on dorsum and flanks add to this remarkable colouration. The flanks below the mantle lack this dark pigmentation and are of a paler colour than the rest.

Neck and head in the dark colour morphs are black as are the ommatophores. The ommatophores are somewhat translucent, and the black ommatophoran retractor can be seen through the integument. No black dotting on the ommatophores is visible. The tentacles are black. In the light colour morphs, the head, neck and the ommatophores are always darker if compared to the body colour.

The keel is of the same colour as the body and thus difficult to see in a crawling animal.

The sole is uniformly creamy yellowish grey in all specimens. In one topotypic specimen, the posterior ends of the lateral sole fields are pigmented with dark grey dots. In two other specimens, the seam of the sole is pale grey with irregularly dispersed, small black dots.

***Mantle structures*.** The pneumostome is positioned on the right side, at ca. ¾ of mantle length, well posteriorly of the middle of the mantle, surrounded by a narrow and almost invisible ring-like structure (Fig. 4F). The visibility of surface structures in living animals strongly depends on the age of the specimen, its condition, and the general air humidity when it is observed. The surface of mantle, besides the sinus groove, can be totally smooth (high temperature/humidity and high production of mucus) or very finely crenulated, or a stage between; the sinus groove is completely visible, often accentuated by a dark pigmentation, reaching almost the end of the posterior mantle edge. The slit of the pneumostome runs anteriorly to at least the dorsal edge of pneumostome. The posterior margin of the mantle is not tightly attached to the integument. The posterior free mantle flap covers the anterior integument-tubercles (Fig. 4A) as well as the slit-like transverse openings of the postpallial pocket organ (Fig. 4E). The posterior mantle edge is markedly indented (curved).

***Postpallial pocket organ*.** As in *Tandoniarustica*.

***Integument structures*.** The number of tr of integument, from the slit of pneumostome to the keel does not vary much (*n* = 3; the topotypes only); from tr 15–17). The surface texture and the width of tubercles in live specimens vary depending on their position on the body. The tr do not appear to be strongly divided in several compartments, only few compartments exist. The tr are all finely crenulated, but also can appear to be totally smooth. In all specimens, the keel extends from the posterior margin of the mantle to the end of the dorsum and is entirely smooth. It is not erected, but evenly rounded, sometimes almost flat and not exposed over the dorsal tr.

***Sole structure*.** As in *Tandoniarustica*.

***Measurements*.**lw (*n* = 3): 1.35–2.5 g, ø 1.85 g; tl (*n* = 2): 46 mm and 70 mm; ml (*n* = 2): 17 mm and 22 mm; sw (*n* = 2): 4.5 mm and 5 mm.

Ratio of tl/ml ranges from ca. 46/17 mm to 70/22 mm; ø ml being a little more than 1/3 of tl.

***Mucus*.** The mucus is transparent on body, mantle and sole, and sticky. So far, no coloured defensive mucus was observed.

***Genital organs*.** Atrium short; penis tubular, constricted in the middle; interior penial wall with a prominent transversely oriented fold; distal to the fold a simple penis papilla (Fig. 5B, C); papilla base slightly swollen; penis and epiphallus equal in length and diameter, divided by a constriction, where penis retractor muscle inserts; epiphallus surface with nodular structures primarily on its proximal end.

Vagina shorter than penis; accessory glands entering distally on vagina, close to pedunculus and oviduct, formed by a broad truncus with bundles of tubuli attached; the vaginal walls richly covered with folds forming a zig zag pattern; a prominent torus with a row of acute conical spikes running through the vagina from atrium almost reaching the branching point of the pedunculus of the bursa copulatrix (Fig. 5D, E); pedunculus shorter than vesicle; vesicle elongated, rounded at the tip; oviduct slim and long.

***Spermatophore*.** No spermatophore found in our specimens. It was described and figured by Regteren Altena (1953: fig. 7).

##### Distribution.

*Tandonianigra* is a rarely found species in Switzerland, and all locally constricted to the Sottoceneri, which is the southern part of the Canton of Ticino, including the districts Lugano and Mendrisio. Our series is very small (*n* = 9). All specimens are from Canton Ticino, from a very restricted area around the Monte Generoso only, including the type locality (*n* = 3). For more information refer to chapters Remarks and Discussion.

##### Habitat.

In the summit region of Monte Generoso, *T.nigra* lives in crevice-rich limestone rocks, which are sparsely vegetated. Under dry weather conditions *T.nigra* is rarely found active on the surface, it is hidden in rock crevices, at least during the day. In the summit region, only very dark-coloured animals have been observed so far. In lower altitudes, *T.nigra* is so far recorded in semi-natural deciduous forests. These are various mixed deciduous forests with beech, hop hornbeam, lime, field maple, ash, hazel, common whitebeam, and manna ash (i.e., southern alpine toothwort beech forest, *Cardamino-Fagetum cyclametosum*, hop hornbeam forest, *Orno-Ostryon*). All habitats are relatively shady. At higher altitudes, these forests are relatively dry, at lower altitudes they tend to be fresh to moist. For the snails, retreat sites with a certain soil cover and air humidity are important. In autumn 2021, however, two animals could be observed crawling during the day in relatively high humidity on a crack-rich, largely dry retaining wall. The first wall was on a mortared garden retaining wall on a natural road, the other on a dry stone wall along a road. Both walls are situated in semi-shade and are only exposed to sunlight for a few hours a day. In the forest, *T.nigra* is found under stones, amongst fallen leaves, in rock crevices and on dead wood. During several hikes in the summit region, only single animals were observed in humid conditions and during sunrise.

So far, all records in Switzerland originate from areas with a good calcium supply. The altitudinal range of its habitats spans from 435 m near Rovio to the highest point at 1700 m.

##### Life history.

There are almost no observations on the biology of the species published. *Tandonianigra* feeds on fallen leaves, but possibly also on lichens and detritus. Mating behaviour and reproduction mode are not yet described. A specimen with spermatophore was collected on 30 July 1948 (Regteren Altena 1953). We conclude that the copulation season for *T.nigra* is in July.

##### Remarks.

The author of this species is Karl Ludwig Pfeiffer (1874–1952), who usually published under the name Karl L. Pfeiffer = K. L. Pfeiffer (Zilch 1972).

The most remarkable new finding is the presence of several/additional colour morphs in *T.nigra*. Pfeiffer’s (1894) sketchy description of the “small and slim” single specimen (most probably a juvenile) he had collected in autumn 1893, is rather uninformative. Nonetheless, he described a few interesting details concerning the colouration: “almost entirely deep black, only towards the sole somewhat lighter, with two longitudinal black lines leaving a very fine yellow line between”. The “very fine yellow line” describes the peripodial groove, the first black line a stripe (peripodial fold) just above the yellow line, the second black line must be the edge of the sole. In our topotypes (*n* = 3) such a fine yellow line was missing in two specimens, while in the other one the line was creamy white. The extremely fine black lines are in fact not visible by the bare eye only. Pfeiffer found “the keel as black as the dorsum and mantle”. Wiktor (1987) mentioned that “Pfeiffer thought it can be a melanistic form of *T.rustica*”, but Pfeiffer (1894) did not mention *T.rustica* at all. Wiktor described the colouration based on a single topotypic specimen (Rijksmuseum Leiden no. 988), which also had been examined by Regteren Altena in 1953. This specimen was differing in some respects from the original description by Pfeiffer: “Mantle at first sight uniformly black, in fact at its edges very dense black dots on dirty creamy ground, visible however only in magnification”. The differences in these descriptions already indicated that for this species some intraspecific variation can be expected.

Our recent research on the new additional colour morphs of *T.nigra* at the lower altitudes of Mt. Generoso at Mendrisio, Val Cürta (PM), and from the surrounding of Rovio (pers. comm. K. Lassauer 2020) has confirmed this impression. These pale creamy specimens were first thought to belong to a different, potentially new species, but our genetic assessment evidenced that these are just colour morphs of *T.nigra*. The penis papilla in some pale colour morphs was less fleshy and bulgy than the topotypic adult specimens. The difference in the penis papilla of some lighter colour morphs might be due to their subadult stage. Concerning the anatomy, the nodular structure on the epiphallus has been visible in fully adult but also in subadult specimens. In some cases, the lumen of the epiphallus was filled with transparent, tiny globuli (Fig. 5C) of unknown function.

In each of the Swiss specimens examined, the posterior end of mantle is markedly indented, as it was already described by Pfeiffer (1894). Pfeiffer (1894) found nine tr on both sides of the body. Most probably he counted the number of tr between keel and the edge of the sole, i.e., at mid of the dorsum downwards to the edge of the sole. Wiktor (1987) reported 13 tr from the single specimen examined by him. This result also does not compare to our counts in the topotypes. The tr can also be totally smooth as already reported by Pfeiffer (1894). The high number of tr will always allow differentiating *T.nigra* from *T.budapestensis*, but not necessarily from juvenile *T.rustica* if not taking pigmentation into account. Concluding, the main intraspecific variability can be found in the body pigmentation as well as in the number of tr on the body. Given the few specimens investigated so far, essentially more research is required to cover all variants in this species.

For a rather long period, *T.nigra*, was thought to constitute an endemic species for Switzerland, or it was omitted as a member of the Swiss Fauna (Forcart 1942). Quite recently, Rähle (1997) identified a population of a small black *Tandonia* species from Valle Brembilla, Bergamo, Italy (coll. SMNS) as *T.nigra*. Forcart (1959) identified a sample (NMB 06166a) from Austria, north Tirol as *Milaxsimrothi*Hesse 1923, which subsequently was corrected by Wiktor (1987) to *T.nigra*. We received a specimen identified by G. Nardi as *T.nigra* from Italy, Laxolo, Valle Brembilla, Bergamo (NMBE 561307), which is almost identical with Rähle’s locality. As a result, Nardi (2017) shaped the distribution area of *T.nigra* over a rather wide range in northern Italy. We tried to re-assess the identity of these specimens.

Unfortunately, it was impossible to extract DNA from the specimen from Laxolo (NMBE 561307), and the preservation state of the animal was poor. As a result, there is no genetic evidence that this is the same species as *T.nigra*. It shares some details with the topotypic Swiss specimens, particularly the presence of the penial papilla, the vaginal torus with the spiked papillae, and the epiphallial node field. However, Rähle (1997) found major differences in the genital organs: He did not find a penial papilla (large and well developed in our specimens), and the accessory glands in his specimen were restricted to some unbranched tubes situated around the distal end of the vagina (large, branched bundles of tubuli in our specimens). This is astonishing as both specimens originate from the same locality. Revision of the Austrian sample (NMB 06166a) failed because of its bad preservation status.

Since its description, eight malacologists (CSCF mapserver, May 2022) have recorded this species from the summit of Mt. Generoso. New sites were found by two malacologists from 2005–2021. Given the doubts in identification of the non-Swiss populations, the question whether this is an endemic species for Switzerland or not is not finally answered.

#### ﻿ Tandonia budapestensis

Taxon classificationAnimaliaStylommatophoraMilacidae

(Hazay, 1880)

4501101E-812F-5B8E-8019-76ACB9CF1BEB

Figs 6 , 7E, F

Amalia budapestensis Hazay, 1880, Malakozoologische Blätter, Neue Folge. 3: 37, pl. 1, fig. 1 [Budapest, Festungsberg im königlichen Garten].

##### Type specimens.

Not researched and not mentioned by Wiktor (1987); probably lost.

##### Diagnosis.

Sole with a dark central field; for other character states, refer to the paragraph under *T.rustica*.

##### Description.

***Colouration*.** Living Swiss specimens (*n* = 10) show a dark rusty-brown to dark chocolate-brown colour on dorsum and flanks to the fringe of the sole. The flanks below the mantle are somewhat paler dark brown grey. Dorsum and flanks, if not unicolourous, may show small black spots and stripes concentrated along the tubercle groves. This can be observed only under magnification and in good light.

The mantle sometimes can be darker than the dorsum because of many black dots and irregular black marbling.

The ommatophores are almost black-brown, but sometimes little translucent in good light, so the black ommatophoran retractor can be seen through the integument. The tentacles are of the same colour.

The colour of the keel ranges from dark brown to rusty orange in its full length.

The sole is grey to dark blackish grey, with the central field sometimes being almost black. In all three sole fields, many black, irregularly jagged spots (chromatophores?) exist, which can be seen only under magnification.

***Mantle structures*.** The pneumostome is positioned on the right side of the body at 2/3 of mantle length, well posteriorly of the middle of the mantle.

***Postpallial pocket organ*.** As in *Tandoniarustica*.

***Integument structures*.** The number of tr on the integument, counted from the slit of pneumostome to the keel (*n* = 7; tr 9/10-tr 12, ø tr 10/11). The low number of tr allows in almost all cases to differentiate *T.budapestensis* from small and dark *T.rustica*. Wiktor (1987) found 9–11 tr, which fits to the variation we found in Swiss specimens. The surface texture and the width of tubercles in live specimens vary somewhat depending on their position on the body. The tr are all more or less finely crenulated. Caused by the low number of tr, they are comparatively wide and obviously wider then in *T.rustica*. All tr are divided in several compartments.

In all specimens, the keel extends from the posterior margin of the mantle to the end of the dorsum. It is not projecting but evenly rounded, sometimes almost flat and not much exposed over the dorsal tr. It is entirely smooth.

***Sole structure*.** The sole structure is similar as in *Tandoniarustica*, but the colouration is not uniform and has a dark central field.

***Measurements*.**lw (*n* = 8): 0.4–1.28 g; ø 0.78 g; tl (*n* = 8): 24.6–62 mm, ø 40 mm; ml (*n* = 8): 7–19 mm, ø 12 mm; sw (*n* = 8): 2.5–6 mm, ø 3.7 mm.

Ratio of tl/ml ranges from 24/7 mm to ca. 62/19 mm; ø ml is little more than 1/3 of tl.

***Mucus*.** The mucus lacks any pigmentation on body, mantle and sole, but is extremely sticky. In a few cases mucus of little pale-orange colour may occur in Swiss specimens.

***Genital organs*.** Atrium wide, spherical shaped; atrium wall covered with folds; penis bulged in the centre; penial walls with long folds ending at muscular ring; penis papilla large, simple oval fold around the opening; papilla basis long, distally shrinking in diameter; penis/epiphallus boundary marked with constriction, where penis retractor muscle inserts; epiphallus matching penis in length; epiphallus surface smooth with apical part kinked.

The vagina is extremely short, separated from the atrium by a muscular ring; accessory glands sac-like attached at centre of the vagina; vaginal walls simple; pedunculus of bursa copulatrix large in diameter, longish vesicle; pedunculus wall with longitudinal internal folds showing a zig zag pattern; oviduct slim and long.

***Spermatophore*.** The spermatophore was described and illustrated by Wiktor (1987: figs 114, 115).

##### Distribution.

*Tandoniabudapestensis* is an introduced species and not commonly found in Switzerland. Our series are small (*n* = 10), and all originate from Cantons Bern, Lucerne, and Zurich. It is rather strictly nocturnal, but occasionally occurs under very wet weather conditions also during the day (pers. obs.). It is a quite inconspicuous slug, and thus only rarely found. The relatively few records at CSCF hardly reflect the state of occurrence in the country, and it is assumed that the species is widely overlooked by Swiss malacologists. It is apparently a lowland species and has not yet reached higher altitudes. This coincides with observations on this species in Bulgaria.

**Figure 6.**
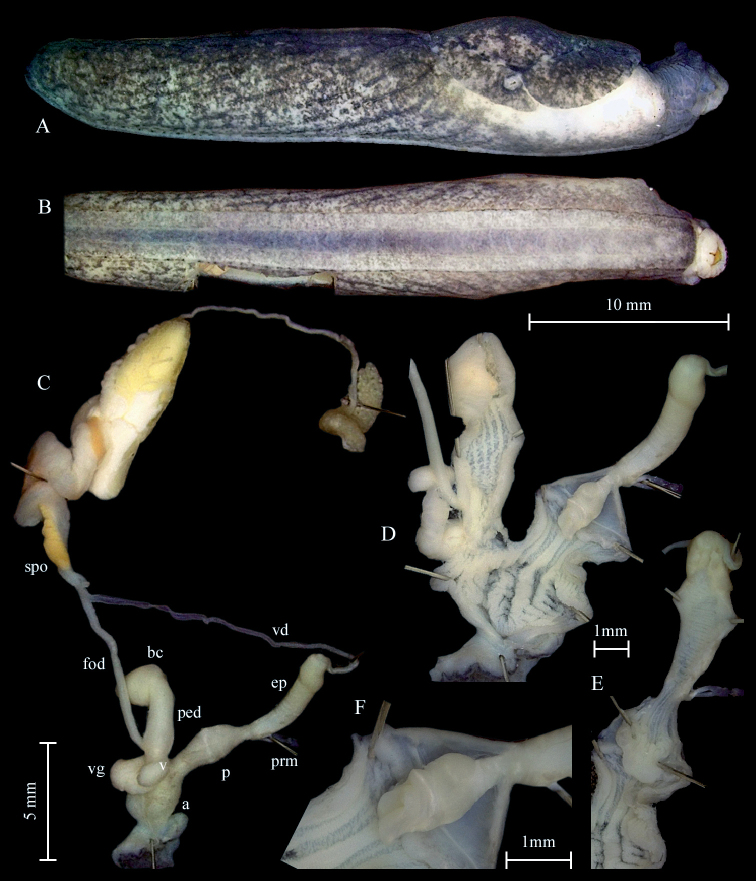
*Tandoniabudapestensis*. Sequenced specimen NMBE 564562, Zurich, Wollishofen. **A** lateral right view **B** ventral view **C** situs of genitalia **D** distal genitalia opened to show inner structures **E** opened penis papilla and epiphallus **F** penial papilla. Abbreviations: a = atrium; bc = bursa copulatrix; ep = epiphallus; fod = free oviduct; p = penis; ped = pedunculus; prm = penis retractor muscle; spo = spermoviduct; v = vagina; vd = vas deferens; vg = vaginal gland.

**Figure 7.**
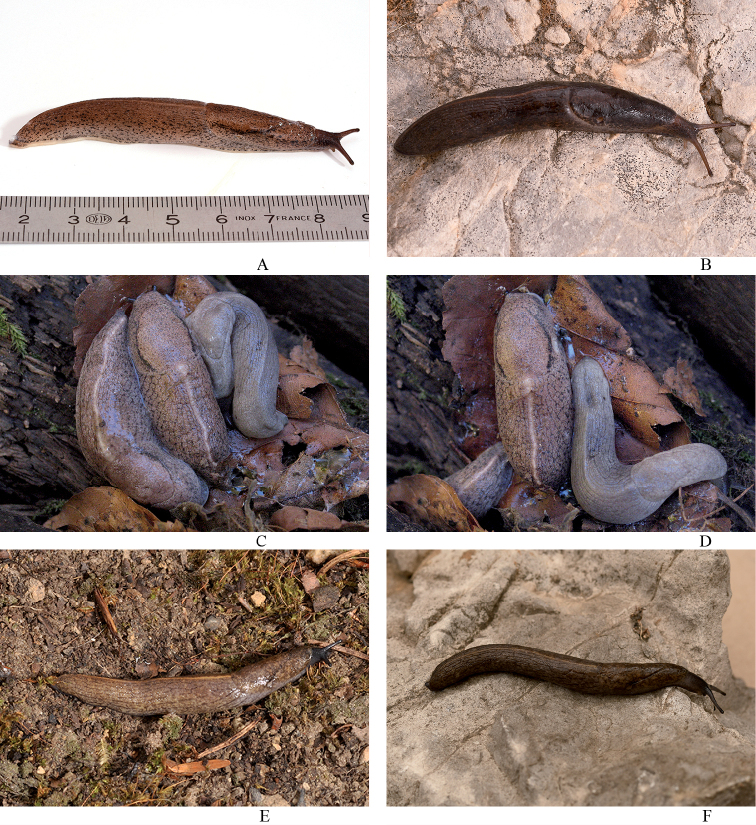
Living specimens of *Tandonia*. **A, B***Tandoniarustica***A** sequenced specimen NMBE 568974, Wartau, Trübbach **B** sequenced specimen NMBE 568973, Rovio, Sovaglia **C, D***Tandoniarustica* (two mottled specimens) meeting a pale colour morph specimen of *Tandonianigra*, Val Cürta, Mt. Generoso **E, F***Tandoniabudapestensis***E**NMBE 571881, Roggwil, **F**NMBE 571882, Meggen.

##### Habitat.

All Swiss specimen were exclusively found in urban areas (anthropogenic habitats) in house gardens, along an old city wall, and close to a small brook with trees and shrubs. The population along the old city wall of Lucerne was checked several times over the years and seems to be stable.

##### Remarks.

We found some Bulgarian specimens ranging from unicoloured yellow to almost completely black. Such colour morphs might be expected in the future in Switzerland, too.

#### ﻿ Milax

Taxon classificationAnimaliaStylommatophoraMilacidae

Gray, 1855

B43DBBFB-50D0-5730-B631-A352D4837B75

##### Diagnostic features.

Atrial accessory glands present¸ atrium with internal stimulator (Wiktor 1987).

#### ﻿ Milax gagates

Taxon classificationAnimaliaStylommatophoraMilacidae

(Draparnaud, 1801)

1CE3E188-2D2E-57D5-83B4-3ECF06E49817

Limax gagates Draparnaud, 1801, Tableau des mollusques terrestres et fluviatiles de la France: 100 [presumably near Montpellier fide Wiktor 1987: 202].

##### Type specimens.

Not investigated and probably do not exist; not mentioned by Wiktor (1987).

##### Swiss specimens examined.

NMBE 510286, Bern, Vechigen, in a garden, July 1970, leg. M. Wüthrich; other record: NMBE 561736, Jura, Porrentruy, in a garden, August 1985, leg. Rüetschi (specimen dried up).

##### Description.

***Colouration*.**Wiktor (1987) describes *M.gagates* as uniformly greyish to blackish, except for the flanks being lighter and always lacking spots. However, our specimen from Vechigen has a beige basic colouration with grey-brown blurred dots covering the mantle and dorsal part of the slug. On the flanks, mainly the longitudinal tubercules are coloured by small brown dots, which results in a rather striped than reticulated appearance (Fig. 9A, B).

The mantle has the same colouration as the dorsum. Above the pneumostome, along the horseshoe-shaped sinus groove, is a prominent grey band.

The neck and head have a darker colouration compared to the body and are uniformly grey coloured without dots.

The keel is also beige, but lighter coloured compared to the rest of the body.

According to Wiktor (1987), the sole varies from grey to blackish, with darker lateral zones and lighter central zone. In the Swiss specimen, the sole shows the same colour as the body, but small, brown-grey dots are spread over the whole sole with a strong accumulation in the central zone. Thus, it seems that the lateral zones are lighter if compared to the central one (see Fig. 9C).

***Mantle structure*.** The pneumostome is positioned slightly posteriorly to the centre of the mantle. The sinus groove is well visible in this preserved specimen.

***Postpallial pocket organ*.** As described in *T.rustica*.

***Integument structure*.** The tr are not countable, most likely because of the preservation of the specimen. The tubercules are large, similar to *T.budapestensis*, and not small as in *T.nigra* and *T.rustica*.

The keel is elevated over the neighbouring tubercules only close to the mantle, and flattens towards the posterior end.

***Sole structure*.** The sole structure is like in the described *Tandonia* species (see *T.rustica*), but instead of a uniform colouration, the sole is lighter on the lateral zones and more pigmented on the central zone.

***Measurements*.** The measurements were done on the preserved specimen NMBE 510286. tl = 30.8 mm; sw = 4.1 mm; ml = 9.8 mm. This results in a ml/tl ratio of ~ 1/3.

***Genital organs*.** Atrium short, spherical; accessory atrial glands are attached centrally to the atrium with several coiled tubules (Fig. 9E, arrow); inside the atrium, a short, pointed but flat stimulator (Fig. 9F, arrow) existing; penis rather long with a constriction in the middle; distal bulb containing penial papilla; penis retractor muscle attached at penis-epiphallus boundary; epiphallus tubular, widened distally; vas deferens entering asymmetrically on epiphallus.

Vagina shorter than penis; pedunculus broad and equal in length to vesicle; vesicle wider than pedunculus, spherical; female oviduct slender and long.

***Spermatophore*.** The spermatophore was illustrated and described by Wiktor (1987: fig. 72).

##### Distribution.

This species is widespread in the western Palaearctic, in Portugal and parts of Spain, France, United Kingdom, etc. (see Wiktor 1987: map 3). Many of the existing populations are considered to be introductions; the original distribution remains unknown. Meanwhile, the species reached an almost global distribution.

**Figure 8.**
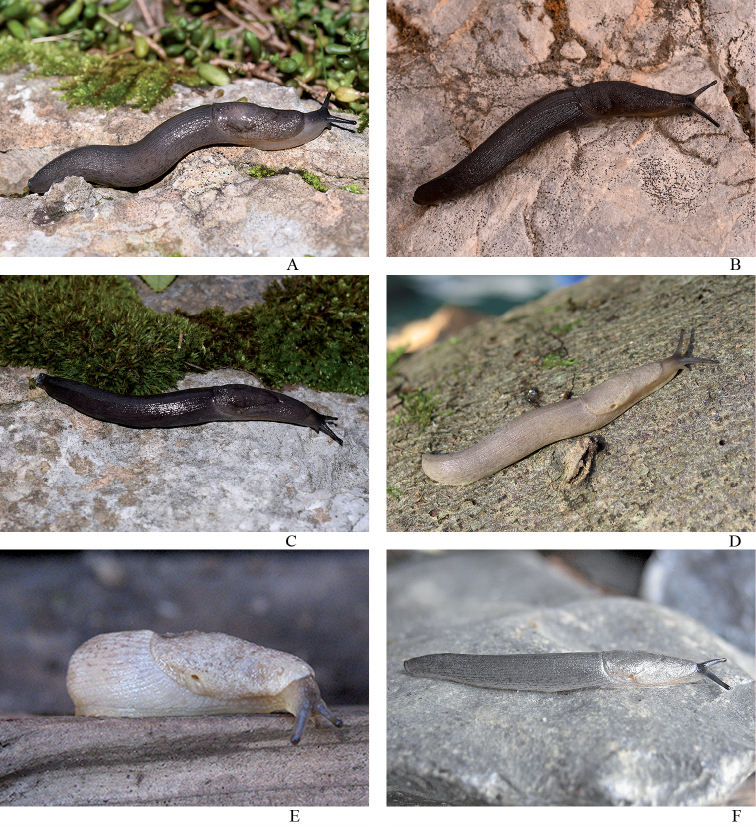
Colour variations of *Tandonianigra*. **A** greyish morph from Rovio, waterfall at Botto, NMBE 568980 **B, C** topotype from summit area; NMBE 569047 **D** Mendrisio, Val Cürta, NMBE 570972 **E** Capolago, Val Cürta, NMBE 570971 **F** Rovio, Sasso Piatto, NMBE 570974.

**Figure 9.**
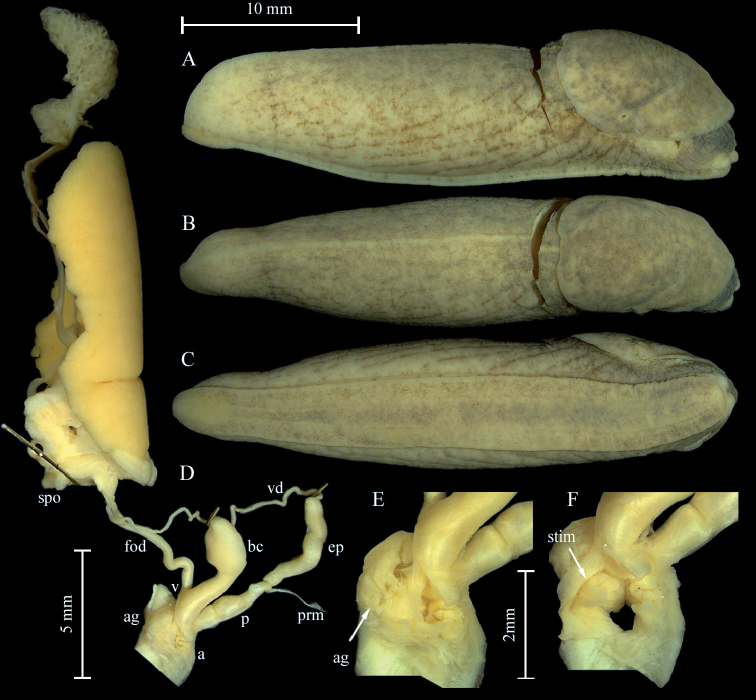
Anatomy of *Milaxgagates*. **A–F**NMBE 510286 **A** lateral view right **B** dorsal view **C** ventral view **D** situs of genitalia **E** accessory atrial glands attached with coiled tubules on atrium **F** stimulator inside the atrium. Abbreviations: a = atrium; ag = atrial gland; bc = bursa copulatrix; ep = epiphallus; fod = free oviduct; p = penis; ped = pedunculus; prm = penis retractor muscle; spo = spermoviduct; stim = stimulator; v = vagina; vd = vas deferens.

##### Remarks.

The specimen from Vechigen is provisionally identified as *M.gagates*. The single animal depicted in Fig. 9 shows the general anatomical character states of this species; however, in our animal, the penis is considerably longer than in the specimen illustrated by Wiktor (1987: fig. 68). A comparison with all other *Milax* species yields no match; the only species, which might be present in the area is *M.nigricans*. This species differs by having a short epiphallus, and long tubules connecting the atrial glands with the atrium. Another potentially reliable distinctive character state can be found in the atrial stimulator: With only a few spines in *M.gagates*, and with lots of spines in *M.nigricans* (see Wiktor 1987; Hutchinson and Reise 2013). However, the stimulator in our specimen was completely reduced and no detailed structures were discernible. Unfortunately, no DNA could be extracted from the rather old specimen from Vechigen.

The very small specimen from Porrentruy was not only completely dried up and completely bleached but turned out to be a young juvenile without any sign of a genital pore. In fact, juvenile specimens cannot be determined on species level from exterior characters, when genital anatomy is not developed. For this reason, we suggest considering this record as *Milax* sp. rather than *M.gagates*.

Finally, there is a specimen housed in the collection of the Muséum d’histoire naturelle de la Ville de Genève, which had been identified as *M.gagates* (MHNG-MOLL-138933, Geneva, November 1968). The specimen was already dissected prior to our investigation and damaged. The investigation of its genital organs yielded no definite result on its specific identity; it certainly is a species from the Milacidae.

## ﻿Discussion

In this study we discuss four species of Milacidae recorded from Switzerland. Three are widely distributed in Europe like *T.rustica*, *M.gagates*, and *T.budapestensis*, while *T.nigra* is considered to be a small range endemic species for the Central Alps. Regardless, the group is badly underreported in Switzerland, and some of the information provided here is new to science (e.g., finding the first spermatophore for *T.rustica*). Usually, the descriptive details provided here are taken from Swiss specimens investigated by the authors; hence, other colour morphs of the respective species may occur elsewhere. A general character analysis embracing specimens from the entire distribution area of a species must be postponed.

Comparing the anatomical character states between *T.rustica* and *T.kusceri*, some discrepancies can be found between our results and those of other authors (Wiktor 1987; Korábek et al. 2016; Turóci et al. 2020) who claim that *T.rustica* is characterised by a pointed vesicle of the bursa copulatrix and a straight epiphallus threefold the length of the penis (Wiktor 1987: 288). In contrast, *T.kusceri* is considered to have a rounded vesicle and a heavily coiled epiphallus 5–6 times the length of the penis (Wiktor 1987: 259). Our sequenced specimens (Fig. 3) unambiguously cluster with all *T.rustica* samples from alpine areas of Switzerland, France, and Italy. Our Fig. 3A–C displays the classical *rustica* conditions in the morphology of its genital organs. This is contrasted by a specimen from Goldswil (NMBE 564708), which resembles the *kusceri* conditions. These observations raise the question of the reliability of such character states, which ideally should be based on investigation of topotypic specimens. Is the shape of the vesicle a fixed trait? Functioning as a receptor of the spermatophore one can expect that the form of this organ may vary according to 1) the maturity of the individual, 2) the form of the spermatophore, and 3) the actual sexual stage the animal reached at the moment of preservation (pre-mating, spermatophore intact inside the vesicle, or spermatophore already digested). Therefore, some variability of the shape should be conceded. For example, Turóci et al. (2020: fig. 7) illustrated a rounded elongate vesicle for *T.rustica*, and the claimed “apically pointed” trait is not visible for us. Obviously, the shape of the vesicle undergoes some variation, and thus cannot strictly be used for species discrimination. It remains to be investigated on how many specimens Wiktor based his firm statement for *T.rustica* (his specimen(s) came from Poland); a comparative study based on several animals from several populations should be performed to clarify this. Similarly, the shape of the epiphallus may depend on the maturity status of the specimen. A coiled epiphallus may be found shortly after the ejection of the (often coiled) spermatophore, and probably explains the differences in our specimens depicted in Fig. 3A–C and Fig. 3E–G. Wiktor (1987: 243, fig. 132) provided a sketched drawing of the penis-epiphallus boundary, where he found two papillae in a row for *T.cristata* (Kaleniczenko, 1851). Here, the penial retractor attaches at a constriction of the “epiphallus”, which is internally accompanied by a first papilla, and then followed by a second papilla pointing into the atrium. Browsing through his anatomical drawings and the position of the papillae, it seems as if some species have a “twin” system, and others have only a distal papilla. Our Fig. 3G shows exactly such a twin case in a specimen, which genetically turns out to be *T.rustica* (see arrows). Obviously, we are far from understanding the morphological details of the male copulatory system of *Tandonia* species, so many more traits await detection and evaluation as to whether they are suitable for species recognition or not. Finally, it must be noted that our use of the names *T.rustica* and *T.kusceri* needs to be corroborated by inclusion of topotypic specimens in future studies. There are no genetic nor detailed morphological data available for animals from Angers (*T.rustica*) nor from Niş (*T.kusceri*), which is prerequisite for the correct application of the names to a biological entity. The fact is that there are two genetically separated clades as proven by Korábek et al. (2016) and Turóci et al. (2020), and which could be confirmed by our study.

We could show that the small and dark specimens of *Tandonia* species from the Alps east of the Monte Generoso, from Monte Baldo, the Bergamasque Alps, north Tirol in Austria, and elsewhere are still in need of a very careful revision. Here, a genetic investigation is pending targeting at taxa such as *T.simrothi* (Hesse, 1923), eventually also *T.baldensis* (Simroth, 1910). Wiktor and Milani (1995) and Nardi (2011) recorded *T.simrothi* from the area of Brescia and further east and northeast, indicating the potential confusion of these species with *T.nigra* as both species are small in size and usually overall darkly pigmented. The newly described brighter colour morphs in *T.nigra* indicate that such a survey should not be restricted to “small and black species”. Otherwise, we potentially overlook existing biodiversity. In fact, Switzerland still has a high responsibility for *T.nigra*, which may be a true endemic of the Mt. Generoso area.

*Tandoniabudapestensis*, an invasive species, was first recorded for Switzerland in 1934 (Forcart 1942; NMB no. 4580a & b). This species can easily be detected and identified. It is known as a potential pest species, but fortunately, records for Switzerland are extremely rare. This might be explained by a bias because malacologists rarely investigate synanthropic sites, and thus the recording has a methodologically biased gap. We consider this species as still significantly underreported for Switzerland, and probably also elsewhere.

*Milaxgagates* is also considered an introduced species and was recorded only three times for Switzerland. Each time, only a single specimen was found. Probably this species is introduced to Switzerland from time to time (Rüetschi et al. 2012); however, as far as we know, no permanent population has been established in the country so far.

For future research we recommend following the preservation procedures for slugs as described above. Perfect samples should be accompanied by photographs taken in the field. Juvenile and subadult specimens should be raised until reaching maturity prior to preservation.

Barcoding and species delimitation such as ASAP can be helpful tools when it comes to the question of species identification, abundance, and distribution. In this study, we found that many characters which were thought to be diagnostic for a specific species have been shown to be intraspecific variables. In some cases, this variability of one or more characters may even overlap with other species, as it is the case for the anatomy of *T.rustica* and *T.kusceri*. In this specific case, a genetic study can help to distinguish those species.

## Supplementary Material

XML Treatment for Milacidae

XML Treatment for Tandonia

XML Treatment for Tandonia rustica

XML Treatment for Tandonia nigra

XML Treatment for Tandonia budapestensis

XML Treatment for Milax

XML Treatment for Milax gagates

## Supplementary materials

Supplementary material 1 Table S1 Data type excel file Explanation note Genetically and/or morphologically investigated specimens. https://binary.pensoft.net/file/725592This dataset is made available under the Open Database License (http://opendatacommons.org/licenses/odbl/1.0/). The Open Database License (ODbL) is a license agreement intended to allow users to freely share, modify, and use this Dataset while maintaining this same freedom for others, provided that the original source and author(s) are credited.Vivianne M. Schallenberg, René Heim, Ulrich E. Schneppat, Peter Müller, Jörg Rüetschi, Eike Neubert
